# Misuse, Abuse and Medication Errors’ Adverse Events Associated with Opioids—A Systematic Review

**DOI:** 10.3390/ph17081009

**Published:** 2024-07-31

**Authors:** Moa Gustafsson, Vítor Silva, Carolina Valeiro, João Joaquim, Florence van Hunsel, Cristiano Matos

**Affiliations:** 1 Department of Pharmacology, Institute of Neuroscience and Physiology, The Sahlgrenska Academy, University of Gothenburg, 41390 Gothenburg, Sweden; moa.gustafsson98@icloud.com; 2 Unidade Local de Saúde de Coimbra, EPE, 3004-561 Coimbra, Portugal; vahsilva@hotmail.com; 3 Eu2P European Programme in Pharmacovigilance and Pharmacoepidemiology, University Autónoma de Barcelona, 08193 Barcelona, Spain; carolinavaleiro99@gmail.com; 4 Instituto Politécnico De Coimbra, ESTESC-Coimbra Health School, Farmácia, 3046-854 Coimbra, Portugal; jjj@estesc.ipc.pt; 5 Netherlands Pharmacovigilance Centre Lareb, 5237 MH ’s-Hertogenbosch, The Netherlands; f.vanhunsel@lareb.nl; 6 Department of PharmacoTherapy, Epidemiology & Economics, Groningen Research Institute of Pharmacy (GRIP), University of Groningen, 9747 AG Groningen, The Netherlands; 7 QLV Research Consulting, 3030-193 Coimbra, Portugal

**Keywords:** opioids, adverse drug reactions, medication errors, abuse, misuse

## Abstract

Opioids are the strongest analgesics available and are crucial in the treatment of acute and chronic pain. The line between these critical medications and how they are used beyond standard therapeutics in cases such as abuse, misuse, and medication errors needs to be understood, as it affects their safety, efficacy, and manner of use. The aim of this systematic review was to identify what is known about the adverse events resulting from the abuse, misuse, and medication errors associated with opioid use. A systematic search was conducted in the PubMed^®^, Scopus^®^ and, EBSCO^®^ databases to retrieve studies from the inception to December 2023 reporting abuse, misuse, and medication errors associated with medicinal opioid use. Two authors independently screened titles and abstracts and full text according to eligibility using Covidence^®^ software. Full articles were examined by two independent reviewers, and disagreements were resolved by a third reviewer. The risk of bias was assessed by the JBI’s critical appraisal tools. A total of 934 articles were screened by their title and abstract. Then, 151 articles were selected for full text screening. Of these, 34 studies were eligible for inclusion in this review. The included studies varied significantly in their population sizes, ranging from 9 individuals to 298,433 patients, and encompassed a diverse demographic, including all ages and both sexes. The studies consistently reported a range of adverse events associated with opioid use. Fentanyl, morphine, oxycodone, tramadol, and hydrocodone were frequently implicated. The data heterogeneity in this field resulted in challenges in drawing conclusions. The review highlights that some opioids, particularly fentanyl, morphine, and oxycodone, are frequently associated with preventable adverse drug reactions, abuse, and medication errors, underscoring the need for robust preventative measures and ongoing research to mitigate opioid-related harm.

## 1. Introduction

Every day, millions of people make use of medicines, some only occasionally, others on a daily basis. While medicines are fundamentally designed to benefit health, their use also involves risks [1,2,3]. Despite rigorous assessments of their quality, safety, and efficacy by regulatory bodies, to ensure a favorable benefit-risk balance at the time of authorization, adverse drug reactions (ADRs) remain a significant concern [1]. ADRs can range from mild to severe, potentially resulting in hospitalization or, tragically, death [4]. A comprehensive review in Europe highlighted that approximately 3.6% of all hospital admissions are due to adverse reactions and about 10% of patients experience an ADR during their hospital stay [5], both children and adults [6,7].

**Figure 1:**
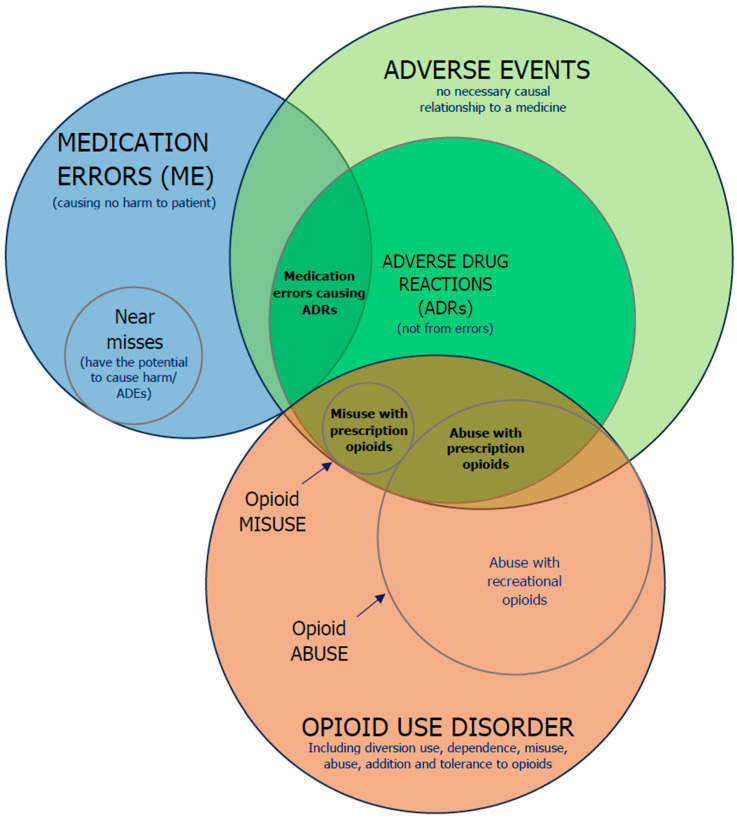
A Venn diagram showing the relation among adverse events, ADRs, medication errors and opioid use disorder; the sizes of the plots do not reflect the relative frequencies of the events illustrated. Illustration supported on the research of previous authors [14,15,16,17,18].

Different definitions of ADR can be found in the literature [8,9]. The WHO defines an adverse drug reaction as “a response to a drug that is noxious and unintended and occurs at doses normally used in man for the prophylaxis, diagnosis or therapy of disease, or for modification of physiological function” [8,10]. Although, in Europe, the definition is actually broader [11], with the European Medicines Agency (EMA) defining an adverse drug reaction as “a noxious and unintended response to a medicine”. This definition was applied in European Union after the change in Pharmacovigilance legislation in 2012 due to the implementation of the Directive 2010/84/EU [11]. The term adverse reaction started to not only include effects from authorized use of the medicinal product and recommended doses but also reactions occurring in different patterns of use (e.g., overdose, abuse, misuse, off-label use, medication errors, or use of falsified products) [11,12]. Abuse of a medicinal product can be defined as a “persistent or sporadic, intentional excessive use of medicinal products which is accompanied by harmful physical or psychological effects”, misuse of a medicinal product is defined as “situations where a medicinal product is intentionally and inappropriately used not in accordance with the terms of the marketing authorization”, while medication error can be defined as “an unintended failure in the drug treatment process that leads to, or has the potential to lead to, harm to the patient” [13]. The relationship between these definitions are represented in a Venn diagram in Figure 1.

### Opioids

Opioids are agents which binds to the opioid receptors in the human body [19]. They are classified as the strongest painkillers and play an important role in managing short- and medium-term pain, but their use for long-term pain is debatable [20]. Opioid receptors are G protein-coupled receptors that mediate the effects of opioids, both endogenous (like endorphins) and exogenous (such as morphine and fentanyl) [21]. The main types of opioid receptors are mu (μ), delta (δ), kappa (κ), nociceptin/orphanin (N/OFQ), and zeta (ZOR). Mu receptors primarily provide pain relief and euphoria; delta receptors are involved in pain relief and mood regulation; kappa receptors produce pain relief but can also cause dysphoria; nociceptin receptors modulate pain and influence anxiety and memory [21,22]. The response can be agonistic, partial agonistic, or antagonistic, but the majority of opioids used in clinical settings elicit an agonistic response, resulting in analgesia [19].

ADRs associated with opioids include respiratory depression, constipation, nausea, vomiting, abdominal pain, bloating, cramping, sedation, confusion, hallucinations, hyperalgesia, central sensitization, pruritus, analgesic tolerance, and addiction liability [19,23,24,25,26], which can affect patients’ quality of life leading to discontinue opioid treatment and, therefore, inadequate pain control [27]. The administration route also determines the pattern of ADRs associated with each opioid. Different administration methods, such as oral, intravenous, transdermal, and intrathecal, can significantly influence the onset, intensity, and type of ADRs experienced by patients [28]. Intravenous administration is more susceptible to the development of ADRs, unlike intramuscular and subcutaneous routes [29]. However, the long-term effectiveness of opioids in managing pain is a topic of debate, with concerns raised about their efficacy over extended periods [20,30].

Long-term use of opioids has been found to cause tolerance that reduces the effectiveness of such drugs, leading to shorter opioid duration of action with repeat administrations [31,32,33]. This tolerance, which is characterized by a decrease in the potency of the drug with repeated administration, is a significant concern with long-term opioid therapy [31], contributing to challenges in the management of chronic pain. Furthermore, prolonged use of opioid therapy has shown a connection with increased possibility of later opioid dependence and overdose without improving functional status [34].

In the past two decades, nearly 600,000 people in the USA and Canada died from opioid overdose events [35]. The opioid crisis in the U.S. has been one of the biggest public health threats in recent decades, with more than 500,000 deaths since 2005 [36], significant reductions in life expectancy, and the development of secondary health conditions including infection, sleep, and affective disorders [37,38]. This crisis developed over the years from the overprescribing of pain relievers and progressed through different stages, from heroin to highly potent synthetic opioid use, such as fentanyl [39]. These substances have exacerbated the epidemic, highlighting the crisis’s dynamic nature and the necessity of addressing polysubstance use [40]. As substances like fentanyl become more common, the risk of overdoses grows, highlighting the urgent need to improve prevention and treatment efforts that address both opioid use and its overlap with other drug use [40].

The ongoing crisis increases mortality but also inflicts broad medical, social, and economic hardships. It was estimated that 1.2 million people might die from opioid overdose by 2029 [35,41]. Recent studies from USA shows that 4.4% of the patients that were prescribed opioids misused them [42], and that in 2020, approximately 9.5 million people misused opioids. Similarly, the European continent has also experienced a rise in opioid use, with misuse and abuse leading to a significant increase in admissions to substance abuse treatment programs and deaths from overdose [43]. Additionally, research has emphasized the importance of estimating the healthcare burden of prescription opioid abuse in European countries in order to develop effective strategies for managing opioid-related issues [44].

In this sense, the aim of this systematic review was to identify what is known about the adverse events resulting from the abuse, misuse, and medication errors associated with opioid use.

## 2. Methods

The protocol of this systematic review was registered in the International Prospective Register of Systematic Reviews (PROSPERO registration number CRD42022358490). The review was structured followed the Preferred Reporting Items of Systematic Reviews and Meta-Analyses (PRISMA) guidelines [45]. The complete PRISMA checklist is available in Table S1.

### 2.1. Search Strategy

A literature search was conducted from inception to December 2023 in the following databases: PubMed^®^, Scopus^®^, and EBSCO^®^. The search strategy is presented in Supplementary Materials Tables S2–S4. All studies were extracted and exported to Covidence ^®^ software (Covidence Systematic review software, Veritas Health Innovation, Melbourne, Australia. Available at www.covidence.org) for screening, review, and data extraction. To reduce the risk of overlooking relevant articles, the reference lists of identified relevant articles were reviewed, and an additional search was conducted using similar search terms.

### 2.2. Eligibility Criteria

Studies about opioids that involved AEs and either abuse, misuse, or medication errors were included. There was no restriction on age, sex, or geographic location. Systematic reviews, preclinical studies, and studies without information of abuse, misuse, or medication errors related to opioids were excluded. Studies not authored in English or that were not possible to translate with clarity were also excluded.

### 2.3. Strategy for Data Synthesis

**Figure 2:**
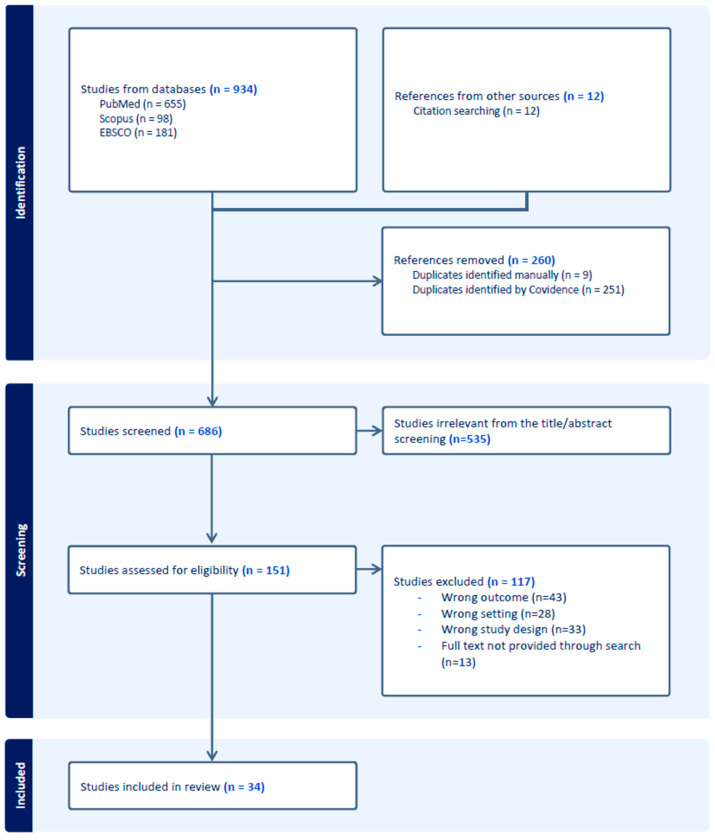
Preferred reporting items for systematic reviews and meta-analysis flow diagram.

The Covidence^®^ software (https://www.covidence.org/) was used to screen, select, and extract data from the articles. Covidence^®^ was chosen because it improves evidence synthesis by enhancing the efficiency of the systematic review. The software mirrors the multiphase review process, including data extraction, directly in its design [46]. Imported duplicates were automatically removed by the software. A title and abstract screening were performed by two independent reviewers (MG and VS), and disagreements were resolved between the two parties or by discussion with a third reviewer (CV). The included articles from the abstract and title screening were subjected to a full text review. Articles were excluded based on criteria such as incorrect outcome, incorrect study design, incorrect setting, or because full-text was not possible to obtain. The data extraction was performed according to the data extraction table (see Supplementary Materials Table S5) by two authors (MG and VS) and reviewed by a third author (CV). Figure 2 represents the preferred reporting items for systematic reviews and meta-analysis flow diagram.

### 2.4. Quality Assessment

The quality of the 34 included studies were assessed by using the JBI’s critical appraisal tools. JBI is an international research organization which develops and delivers evidence-based information, software, education, and training designed to improve healthcare practice and health outcomes, including the critical appraisal tools to assess the methodological quality of a study and to determine the extent to which a study has addressed the possibility of bias in its design, conduct and analysis. The JBI tool for case series was applied to the 25 case series studies. The JBI tool for cohort studies was applied to the nine cohort studies. The result of the quality assessment is presented in the Supplementary Materials—see Tabled S6 and S7. Based on the risk of bias score, no paper was rejected.

## 3. Results

In the 34 included studies, the research population ranged from 9 [47] to 298,433 patients [48]. The studies included different age ranges, including pediatric population [49,50,51,52], elderly [53,54], or all ages [28,35,48,55,56,57,58,59,60,61,62]. Other references used that did not specify the age of their patients [63,64,65,66]. In one publication, abuse was the only exposure investigated [60]. In the remaining studies, medication errors were either the only topic of investigation [49,50,52,53,55,58,62,63,64,66,67,68,69,70,71,72,73,74,75,76] or were combined with abuse [28,56,57,60,65,77,78,79,80,81,82,83,84] or unintentional misuse [48]. Additional issues such as addiction, AEs [28,54,65,78,83,84,85], and overdose were also addressed in different studies [35,64,65,79] (Table 1).

### 3.1. Prevalence of Opioid Abuse

The prevalence of opioid abuse varies significantly across different regions and healthcare settings, reflecting the complexity and scope of this subject globally [83,84]. Studies included in the review cover different regions such as the USA [35,48,52,57,59,60,62,64,66,68,69,75,77,79,80,82,84], Europe [28,49,50,55,56,57,58,63,65,67,78], Australia [54,73,74], and Canada [51,83], while countries from Asia, Africa, and South America were not included. A study reported a prevalence of opioid prescription in primary care settings from 0.6% to 0.8% [86], as well as a lower rate of abuse compared to other settings, possibly due to more controlled prescription practices and closer patient monitoring in primary care [86]. Lower abuse rates in primary care settings were inferred from general patterns of prescription and abuse [65] when compared with specialized clinics due to opioid-related AEs in emergency departments [75,76]. In contrast, in clinical settings, the prevalence of opioid abuse is reported as significantly higher, ranging from 8% to 16% [86], with patients often prescribed opioids for chronic pain management, potentially leading to higher risks of dependency and misuse [86].

The abuse of opioids goes far beyond their prescription use: the use of opioids without a prescription significantly increases the rate of abuse in the population, as the lack of adequate medical monitoring can lead to misuse and dependence [77]. Different authors highlight adverse events arising from abuse and diversion [28], including overdose deaths [48,75,87]. This abuse uses opioids in potentially harmful ways but without the intention of achieving a euphoric effect (misuse), and those who use opioids intentionally to achieve such effects (abuse) [28].

The most commonly abused opioids have changed over time and vary depending on the country [56,77,83]. Tilidine was initially the most commonly abused opioid in Germany, but over time, tramadol became more predominant [56]. Tilidine has faced concerns about its potential for abuse from early on [88] and is no longer available in the market in some EU countries [89]. In different European countries, the potential of abuse was highlighted [89,90], with Belgium withdrawing it due to issues related to its abuse [90]. In Canada, fentanyl emerged as the principal opioid of abuse, particularly when combined with benzodiazepines, leading to an increase in fentanyl-related deaths since 2012 [83]. In the United States, regulatory changes in 2014 resulted in a reduction in supratherapeutic ingestions of hydrocodone/acetaminophen and an increase in codeine/acetaminophen ingestions from 2015 onward [77]. In this review, the most frequently mentioned opioid in the list was morphine (16 mentions), followed by fentanyl (15 mentions) and oxycodone (12 mentions). Other opioids included hydrocodone, tramadol, and codeine (eight times each), buprenorphine and hydromorphone (seven times), methadone (five times), and tapentadol (two times).

The heterogeneous effects that different opioids evoke could be one of several contributing factors leading to intentional abuse [91], but it seems that drug availability has the biggest influence [92]. A survey from the United States revealed that 94% of respondents claimed they chose heroin over prescription painkillers because it was less expensive and simpler to obtain [93]. Those results are consistent with the previous research, namely that abuse behavior is influenced by availability of the substance [92].

### 3.2. AE Following Medication Errors

Medication errors involving opioids are a significant concern due to their high potential for causing AEs and harm to patients. They were described in 27 studies [28,35,48,49,50,51,52,53,55,58,59,62,63,64,66,67,68,69,74,75,78,80,81,82,83] and different errors were found: (a) errors during prescription, including inappropriate or ineffective prescribing practices and both underprescribing and overprescribing [51,66,80]. Those errors involve issues such as illegibility or unclear electronic entries [50,51,66]; (b) dispensing errors, with the wrong drug or formulation, as well as labeling mistakes [49,53,59,73,74]; (c) administration errors which involve incorrect dosage, route, or timing [55,67,68]; and lastly, (d) monitoring errors with insufficient treatment monitoring and failure in therapy adjustment based on patient response or side effects [67], especially in treatments needing close supervision for efficacy and safety [67]. Medication errors can be caused by both healthcare professionals and patients. Healthcare professional errors include prescribing errors due to illegible handwriting or unclear electronic inputs and administration errors such as incorrect dosage, route, or timing [50,51,55,66,67,68]. Healthcare professionals may also be responsible for monitoring errors when there is insufficient monitoring or failure to adjust therapy based on patient response [67,74]. However, patients themselves can contribute to medication errors by misunderstanding prescription instructions, taking incorrect doses, or failing to adhere to the prescribed use [49,53,58].

The incidence of these errors varies between studies: in a study focused on pediatric anesthesia, opioids were identified as the drug class most responsible for medication errors, with 10 (0.7%) cases of errors identified involving opioids [49]. Andreaggi et al. found that out of 300,985 ADRs analyzed, 3.4% were due to medication errors involving opioids [80]. Cobaugh et al. reported 543 (1.71%) cases of therapeutic errors using opioids among older adults, of which 305 were from morphine and 238 from other opioids, highlighting the hazard resulting from these medications [53]. In the general population, Ni et al. reported 30.3% (*n* = 51,715) of drug overdose reports as involving opioids out of the total number of drug overdose reports in the FAERS database, indicating a high incidence of medication errors [35].

Medication errors involving opioids can lead to severe adverse effects on various organ systems, primarily impacting the respiratory and gastrointestinal systems [49,52,63,65]. Common adverse events with opioids include respiratory depression, with occurrence dependent on the dosage [28,49,74] and identified for different opioids such as alfentanil [49], remifentanil [49], sufentanil [49] tramadol, and morphine [49,65]. Other AEs include gastrointestinal disorders, constipation, nausea, and vomiting, which were reported by Cobaugh et al. among older adults due to therapeutic errors [53] or by Lovegrove et al. among children under the age of six, who found that opioids were the most implicated medication class in emergency department visits involving prescription solid exposures, often leading to gastrointestinal symptoms such as nausea and vomiting [69].

Despite the fact that the opioid crisis is still more evident in the USA, opioid-related problems are perceptible in different countries: the most common substances to cause fatal medication errors in Ireland are cocaine, antidepressants, and benzodiazepines [94]; in England and Wales, anticoagulants and opioids were reported as responsible for almost half of the medication-related deaths (22%) [78].

### 3.3. AEs Following Abuse and Misuse

AEs following opioid intentional abuse or unintentional misuse were described in seven studies [56,57,65,77,80,81,83]. The rates of misuse and abuse depends on the opioid substance observed as well as the setting. Andreaggi et al. reported a total of 22,167 (7.4%) cases of drug abuse [80]. Harris et al. found that abuse and misuse resulting in overdose were found in 25% of cases (*n* = 186) in an emergency department for paracetamol/opioid-containing products [77]. Lorenzini et al. found that 64 patients visited the emergency department due to opioid-related ADRs, with 16 overdoses and 19 (29.7%) cases of abuse or misuse identified [65]. Common opioids involved included tramadol, morphine, and buprenorphine [65]. The use of opioids combined with another medication increases the risk of AE through abuse and misuse [83], e.g., the coadministration of tapentadol with other medications, including opioids, benzodiazepines, antidepressants, and antihistamines, significantly increased the risk of AE [81]. This misuse resulted in significantly higher tapentadol concentrations in postmortem cases than therapeutic levels, indicating potential abuse [81]. Chatterton et al. reviewed 2812 fentanyl-positive cases, with approximately 45% of these cases also involving benzodiazepines [83]. The median concentration of fentanyl in cases involving benzodiazepines was significantly higher than in cases without benzodiazepines, indicating that higher or more potent doses of fentanyl were being abused [83].

The abuse of hydrocodone and oxycodone are frequently highlighted due to their potent effects and widespread availability, leading to significant misuse and associated medication [56,77]. Similarly, methadone and morphine are also related to misuse and abuse [56,57]. Their dual role in therapeutic contexts and high potential for abuse make them a focal point in discussions about opioid misuse [57].

Dependence, withdrawal symptoms, and cognitive impairment are common neurological consequences of long-term opioid misuse [56]. Jobski et al. reported on the neurological effects of opioid dependence and withdrawal, highlighting the significant impact on patients’ central nervous systems [56]. Additionally, Chiappini et al. analyzed AE from major pharmacovigilance databases to detect issues related to abuse, misuse, dependence, and withdrawal of opioids, providing insights into the long-term neurological effects of misuse [57].

### 3.4. Medication Related Deaths

Several studies have documented the occurrence and impact of drug overdoses, focusing on opioids [35,64,65,73,79]. Looking at drug overdose events reported to the U.S. Food and Drug Administration (FDA), opioid analgesics were found to be the most frequently implicated drug class. They accounted for approximately 30.3% of all drug overdose reports from 2017 to 2021, making them the top drug class associated with overdose incidents [35,95]. Heneka et al. documented that opioid toxicity was in 39% (*n* = 7) of these overdose patients [74]. Lorenzini et al. provided detailed numbers of opioid overdoses in their review of emergency division (ED) visits [65]. In a total of 12,470 emergency room visits, overdose cases were identified in 16 opioid users (1.5% of opioid users; 0.1% of total ED visits), accidental (*n* = 8) and deliberated overdoses (*n* = 8) [65]. Seth et al. documented a 21.5% increase in overdose deaths from 2015 to 2016, with synthetic opioids contributing significantly to the rise [79]. In USA, ten states experienced increases of ≥100% in the number of overdoses, with Columbia (392.3%), Illinois (227.3%), and Maryland (206.9%), being the states with the greatest increases [79].

Fentanyl has been extensively documented as particularly contributing to the high number of overdose deaths occurring in non-pharmacological use [68,79]. In 2016, synthetic opioids, including fentanyl, accounted for 30.5% of all drug overdose deaths in the United States, with a 100% increase in the rate of these deaths from the previous year, primarily driven by illicitly manufactured fentanyl [79]. Lorenzini et al. and Gariel et al. have documented respiratory depression due to overdose [49,65].

Some of the remaining articles investigated whether or not the ADRs had major consequences or caused death [80,84], as well as whether or not the ADRs could have been avoided without going into detail about what those serious effects were [78].

Medication-related deaths following AEs were described by different authors [53,68,78,79,81]. Bailey et al. reported 8 deaths out of 51 cases with major serious events in a large number of young children exposed to opioids (*n* = 9179). The study highlighted that nearly all exposures occurred through ingestion (99%) and in the home (92%) and were unintentional, involving medications prescribed for adults in the household, primarily the child’s parents [68]. Opioids accounted for approximately 20% of all medication-related deaths reported in the USA [96].

Chatterton et al. analyzed postmortem blood concentrations of fentanyl and benzodiazepines, linking high concentration levels directly to toxicity and deaths [83].

### 3.5. Preventability of Adverse Events and Medication Errors

According to Culleré et al., 9.3% of all preventable AEs were triggered by use of opiates [67]. Based on three studies focused on opioid-related ADRs, 39% [58] to 64% [64] of them could have been prevented [58,64,73]. Heneka et al. concluded that many errors could be avoided with adequate training and clinical decision support systems [73].

Different studies have explored strategies and interventions aimed at reducing medication errors associated with opioids, particularly focusing on high-risk medications like fentanyl [63,72,97]. Adequate storage and child-resistant packaging measures were highlighted as significant in preventing AE and medication errors [68]. One study highlighted the importance of pictorial prescription in reducing fentanyl drug administration errors, using visual aids in prescribing practices to minimize medication errors related to opioid administration [97]. Another study explored Technology-Based Interventions that introduce smart pump systems to prevent medication administration errors, especially with opioid infusions [65]. The importance of continuous quality improvement and error reporting systems has been highlighted to identify and address medication errors involving opioids [63].

### 3.6. Pediatric Population

Pediatric populations were highlighted in different studies [49,50,51,52,68]. A study addressing medication errors in pediatric anesthesia revealed that opioid analgesics, such as morphine and fentanyl, are frequently implicated in incidents, and are the drug class involved in the most errors; however, they were also the type of medication that was most frequently used [49]. In pediatric care, errors commonly occur through dosing errors, including uncertainty between weight-based calculations and doses actually administered [50,52]. Incidents involving incorrect opioid dosages have led to serious adverse events, including respiratory depression and overdose, which can result in prolonged hospitalization, serious outcomes, or eventually death [49,52]. In Bailey et al.’s study, 9179 children were identified as having been exposed to prescribed opioids [68]. Of these, there were 8 deaths and 43 cases classified as major effects. Most exposures occurred accidentally at home, with nearly all resulting from ingestion of medications prescribed to adults within the household [68].

Hicks et al. indicated that pediatric opioid use must be handled with extreme caution to avoid dosing errors that can lead to overdose or inadequate pain control [52]. To improve safety, the use of standardized protocols for opioid administration, double-checking systems, and advanced training for their professionals in pediatric dosing and opioid management have been recommended [52].

### 3.7. Genetic Polymorphisms Associated with Adverse Events

Based on the studies analyzed, genetic polymorphisms, particularly involving the CYP2D6 enzyme, significantly influence the pharmacokinetics and pharmacodynamics of certain opioids. Lorenzini et al. (2022) highlighted that genetic variations in CYP2D6 can lead to poor metabolizers producing fewer active metabolites, reducing the analgesic effects of opioids like tramadol, codeine, and oxycodone [65]. Moulis et al. (2018) also noted the impact of CYP2D6 polymorphisms, mentioning that ultrarapid metabolizers can significantly alter drug metabolism [50]. Similarly, Day et al. (2016) discussed how codeine, a prodrug requiring conversion to morphine via CYP2D6, exhibits considerable metabolic variability due to these genetic differences, affecting its efficacy as an analgesic [48].

### 3.8. Quality Assessment

The risk of bias of the articles was evaluated using the JBI bias tool for systematic reviews, see Supplementary Materials Tables S6 and S7.

## 4. Discussion

Opioids are considered the strongest analgesics and have an important role in managing short- and medium-term pain, while their role in managing long-term pain is under discussion [28]. Their use is not without risks, as there is a growing concern about the misuse and abuse of opioid analgesics, leading to serious consequences [98].

The pharmacological characteristics of opioids, including their potential to induce euphoria, can lead to recreational use, misuse, and eventually abuse [35]. Chronic use of opioids leads to tolerance, whereby larger doses are needed to produce the same level of effect, and dependence or withdrawal symptoms, which refer to the occurrence of certain effects when the drug is not administered [65,83].

Also, chronic and severe pain patients often cannot receive sufficient pain management treatments and have to depend on opioids: inadequate availability of alternative pain treatments forces patients to depend on opioids, increasing the risk of misuse and dependence [66].

Medication-related problems, such as medication errors, misuse, and abuse involving opioids, are common across different settings, including hospitals and palliative care, as well between different population groups, such as elderly people and children. ADRs associated with opioids include serious outcomes resulting in hospitalizations and deaths, with morphine, fentanyl, oxycodone, tramadol, and hydromorphone frequently implicated [28,65,83,84].

Economic factors contribute to these opioid-related problems, since the high cost of prescribed opioids may push individuals to seek cheaper, illicit alternatives like heroin, further complicating the problem [79]. However, prescribed opioids are readily available and prescribed in large quantities, making them easily accessible for non-medical use [52]. Studies have highlighted that the excessive prescription of opioids could increase the potential for abuse [52,56]. Additionally, the recreational use of prescribed opioids for unauthorized use is a significant issue, leading to widespread community abuse and increasing the risk of dependence and overdose [68,73].

Inadequate prescription practices lead to errors such as incorrect dosages and insufficient monitoring of patients can result in medication errors [49,50,56,76], and ultimately bring patients to misuse or abuse situations. Patients often lack proper guidance on the safe use of opioids, which contributes to inappropriate use of the drugs [77,80]. Furthermore, sociocultural and psychological factors, such as self-medication for emotional or psychological pain and social influence, exacerbate the issue. Individuals may resort to using opioids not only for physical pain relief but also to manage emotional distress, while peer pressure and cultural norms around substance use can drive opioid abuse [49,57].

Dependence and withdrawal problems related to opioids are often described in cases of abuse or chronic use, with authors studies utilizing post-marketing surveillance databases highlighting dependence [28,56,57]. Data from the European Medicines Agency (EMA) EudraVigilance database provide descriptive statistics on opioid dependence and abuse, highlighting variations in reporting completeness and characteristics by different healthcare professionals and consumers [56]. The overall incidence rate of AEs in hospitalized patients was 10.3%, with 51.6% of these AEs being preventable [67]. Opioid dependence can develop quickly, particularly with potent opioids such as fentanyl or oxycodone [56,65]. This dependence is enhanced by the pharmacological characteristics of opioids [35]. Withdrawal symptoms such discomfort, pain, nausea, vomiting, and diarrhea present a challenge for individuals dependent on opioids [56,65]. This underscores the necessity for appropriate medical intervention and support to manage withdrawal and help individuals cease opioid use effectively.

Regarding opioid overdoses, most of the evidence comes from North America, especially the USA and Canada, where the opioid crisis is most pronounced, with studies focusing on both adults and children and analyzing a wide range of opioids [51,68,75,79,83,84], fentanyl being a common cause of overdose [75,84]. Studies from Germany, France, the Netherlands, and Australia also underscore significant opioid-related problems, recurrently noting dependence, abuse, and medication errors [28,49,50,54,56,73,74,81,84]. Studies use surveillance databases like the FAERS [35] and EudraVigilance [57], analyzed patterns of abuse, ADRs, and medication errors, while case reports offer detailed insights into specific opioid-related issues. Raising awareness and educating healthcare professionals, along with rigorous patient monitoring, are essential to reduce the risks associated with opioid use [73,74].

The application of different strategies, such as integrating smart pump information and data quality evaluation to detect and prevent medication administration errors, particularly in the context of opioid infusions [72], would help healthcare providers to further strengthen their medication safety practices and minimize the number of these potent opioid-related medication errors. Effective approaches include thorough education for patients and healthcare providers, stringent monitoring of opioid prescriptions, and improving access to alternative pain management options [67,74]. Implementing these strategies can help mitigate the risks associated with opioid use and improve patient outcomes [48,74].

The types of drugs most included in medication errors were also studied in the articles in this systematic review. However, the results are diverse, with results ranging from concluding that opioids are the drug type that causes the most errors [52] to presenting opioids as the 9th most error-causing drug type [55]. Deaths following the use of opioids were reported in four articles without comparisons to other drug classes [47,55,57,65]. This variation may be due to several factors, including differences in study design, populations examined, and specific healthcare settings. For example, in general medical settings where a greater variety of medications are used, opioids may not be the predominant medication type involved in errors. Understanding the diverse results and contexts presented in studies is important for developing strategies to minimize opioid-related medication errors and improve patient safety in different healthcare settings.

Different strategies could be used to prevent adverse events such as misuse, abuse, and medication errors associated with opioids. For healthcare professionals, education and training on safe opioid prescription practices [99], on the management of opioid-related ADRs, and on the use and implementation of clinical decision support systems can aid in reducing errors and improving prescription and pharmacotherapy for patients using such drugs. Education on the topic should be addressed in their medical curricula and through continuous education campaigns [99,100]. Stricter opioid prescription practices and prescription-monitoring programs have been implemented in order to lower the population exposure to these drugs [100,101]. Other strategies identified in the literature include risk assessment plans and interventions to improve the use of opioid analgesics, education on pain management to reduce opioid prescription, and the implementation of evidence-based primary prevention programs to reduce the demand for opioids [100,101]. Patients with prescribed opioids should be continuously monitored, including regular follow-ups that can help identify and mitigate potential misuse or abuse earlier or disclose an accidental error. The use of pictorial aids for better patient understanding, smart pump systems for controlled opioid administration, and double-checking protocols for dosing (e.g., in pediatrics) can significantly reduce the medication errors associated with opioids. On the other hand, there is major underreporting for medication errors due to several reasons, including fear of legal action, blaming individuals instead of the system, ineffective reporting systems, lack of feedback, and insufficient support for those who commit errors, as well as the absence of a reporting system and lack of awareness about how to report medication errors [102]. Awareness of reporting systems can help to improve the information about medication errors. Finally, raising awareness about the risks of opioid misuse and abuse through public health campaigns can help reduce stigma.

In the USA, the heavy economic burden of opioid abuse, dependency, and overdoses has led to the emergence of this issue as a major priority. Studies have highlighted the substantial costs associated with opioid-related issues, including healthcare expenses and criminal justice costs [103,104].

Regulatory agencies have a responsibility to ensure that drugs on the market have a positive risk–benefit balance. In the US, authors have voiced concerns [105,106,107] that the U.S. FDA was not strict enough in the past in enforcing marketing regulations for opioid drugs, especially since there has been an incredibly steep increase in the use of opioids in the U.S. [108,109]. In combatting issues with opioid addiction, regulatory agencies, such as the FDA, have the authority to approve new and safer formulations of immediate- and long-acting opioid medications, restrict indications, issue Dear Healthcare Professional Communications (DHPCs) and risk minimalization materials, regulate industry promotion, and suspend the marketing approval for products with a negative risk–benefit balance [110]. These are all potential regulatory actions that can be used to make opioid use safer, and the FDA has issued a timeline of the actions they have taken to address substance abuse and overdose prevention [95]. However, in order to let these regulatory actions be impactful, changes in behavior and in the clinical practice of other stakeholders, such as clinicians, patients, and marketing authorization holders, are also needed [95,111,112].

The quality of the studies was assessed using the JBI bias tools for systematic reviews, chosen due to the included articles’ study designs. Five cohort studies received lower scores (<50%), and all of these studies did not adequately identify or address confounding factors, which are essential to validating the reliability of study results. Furthermore, these studies failed to report completeness of follow-up, nor did they employ strategies to mitigate the impact of incomplete follow-up data. There were inconsistencies in how exposures were measured between groups, and it was unclear whether study participants were free of the outcome at the start of the studies. These methodological deficiencies significantly compromised the overall reliability and validity of their results, explaining the lower scores, but none of the studies were excluded due to the large number of not-applicable answers [58,59,64,66,73]. The case series did not report clinical information of the patients in detail, which might make it difficult to draw conclusions about the correlation between the opioid and ADRs. Most of the articles provided clear demographic information, including age range and sex. Ethnicity was reported in some studies, and it has been reported that this can affect drug metabolism, such as the risk of being an ultra-rapid metabolizer for codeine through the CYP2D6 enzyme, which can result in medication errors for this subgroup. However, extensive variability within subpopulations has also been reported, and relying on reported ethnicity alone without pharmacogenomics is unadvisable [113]. Socioeconomic status may influence the number of medication errors, abuse, and misuse, since some studies reported that higher socioeconomic status correlates with better health [70,114].

### Strength and Limitations

The risk of bias of the included studies were generally low (see Supplementary Materials Tables S6 and S7), demonstrating the good quality of the studies included. The heterogeneity of the data is one of the limitations of this study. It makes it difficult to compare results. Additionally, the country information often refers to the database origin, but databases such as FAERS and EMA receive reports from countries other than those represented, which can lead to inconsistencies and duplicated data. Furthermore, different studies often report data from the same databases, resulting in also potential conflicts and duplication of data [115]. These factors can make complex interpretation and reliability of the findings.

The aspects that are investigated in the studies are also limited and some relevant information is not presented or investigated. Medication errors, abuse, and misuse are costly [104], but none of the articles investigated the economic burden impact of those problems. Cost-analysis studies could highlight the importance of prevention and the potential to significantly reduce the economic burden of the opioid pandemic.

Unintentional misuse was investigated in different studies [56,57,65,77,80,81,83]. However, the definition of misuse lacks clarity, being “*situations where a medicinal product is intentionally and inappropriately used not in accordance with the terms of the marketing authorization*” [13]. An unintentional misuse would therefore more likely be a also a medication error as long as it is an error caused by a prescribed drug. If a described misuse is an “*excessive use of medicinal products which*” or “*is accompanied by harmful physical or psychological effects*”, it should be also classified as abuse; however, the definition of abuse is restricted to intentional use, which is controversial [13]. Given the overlaps and ambiguities in the current definitions of misuse, abuse, and medication errors, further clarification is needed to ensure accurate classification and avoid data loss or overlap.

All the studies that made up this systematic review gathered their data from patients who had interacted with healthcare providers. As a result, there is a significant risk of underreporting, both from healthcare professionals [116,117] and from patients [118]. Due to the stigma associated with opioid use, there is also a risk that fewer ADRs will be reported [119]. Patients may also avoid reporting due to concerns about legal repercussions, damage to their reputation, or fear of losing access to future prescriptions [35,65]. Some articles categorized the ADRs that followed opioid use as serious or not without naming the specific ADRs [51,80]. Further to this, there was no other data only concerned with the ADRs brought on by the misuse of opioids. Differences in study designs and countries included help to illustrate the geographic variations in opioid-related problems. Larger study populations and more studies investigating the preventability of such problems would have been required to narrow the spread in this study [57].

This systematic review only focused on published papers regarding opioids in combination with medication errors and/or abuse and/or misuse. Currently, it was not possible to determine which opioid causes the greatest incidence of opioid misuse, abuse, or medication errors.

## 5. Conclusions

In different regions, studies show that opioids are frequently associated with abuse, misuse, and medication errors, indicating a widespread issue. Certain opioids, such as fentanyl, morphine, oxycodone, tramadol, and hydrocodone, are recurrently involved in ADRs, abuse, and medication errors. Fentanyl, in particular, is highlighted for its high risk of abuse and overdose. Many of the reported ADRs, including serious outcomes like respiratory depression, constipation, nausea, sedation, and overdose deaths, appear to be preventable. Overdose deaths are particularly prevalent with opioids like fentanyl, morphine, and oxycodone. Several studies indicate that a significant portion of opioid-related ADRs and medication errors are preventable, which underscores the importance of implementing robust preventative measures and safety protocols. Continued research is essential to better understand the safety profiles of different opioids and develop effective strategies for preventing opioid-related harm, including continuous education, stricter prescribing practices, effective monitoring, and public health campaigns to mitigate the risks of opioid misuse, abuse, and medication errors.

## Figures and Tables

**Table 1:** Summary of the data included in this study.

Author, Year	Country	Study Design	Aim of Study	Number of Reports/ Patients	Age (Years)	Opioid(s) Used	Type of Use: Prescribed Medicines	Type of Use: Recreational/Diversion	Type of Problems Reported
Chatterton et al. (2023) [83]	Canada	Case Study	To determine the concentrations of fentanyl and benzodiazepines in postmortem blood and their impact on toxicity and death.	A total of 2812 detected cases of postmortem fentanyl concentration.	Adults ≥18	Fentanyl.	Not stated	Not stated	Abuse; ADRs; medication errors; misuse.
Harris et al. (2023) [77]	USA	Cohort	To assess the impact of federal regulations on the rates of supratherapeutic acetaminophen-opioid ingestions.	A total of 186 (25%) emergency department encounters related to paracetamol/opioid products.	Adults ≥18	Hydrocodone/acetaminophen; codeine/acetaminophen.	Not stated	Not stated	Abuse; misuse; ADRs.
France et al. (2023) [78]	England and Wales	Case Study	To identify and characterize medicine-related deaths from coroners’ reports to prevent future deaths.	A total of 257 (22%) preventable deaths were reported due to analgesic opioids.	Adults ≥18	Other: not specified.	Yes (63%)	Yes (28%)	Abuse; ADRs; death; medication errors; misuse.
Ni et al. (2023) [35]	USA	Case Study	To analyze and evaluate the scope of drug overdose reports related to specific drugs in the FAERS database, with a focus on identifying the drugs most frequently reported in association with overdose.	A total of 51,715 (30.3%) cases reported of drug overdose with opioids.	All ages	Morphine; oxycodone; hydrocodone; buprenorphine; codeine; meperidine; tramadol.	Not stated	Not stated	Medication errors.
Jobski et al. (2023) [56]	Germany	Case study	To analyze the completeness and characteristics of spontaneous reports of opioid-associated abuse, dependence, or withdrawal from Germany, focusing on the reporter.	A total of 1721 cases included from Eudravigilance.	All ages	Tilidine; tramadol; morphine; fentanyl; oxycodone; oxycodone/naloxone; hydromorphone; tapentadol.	Not stated	Not stated	Abuse; dependence; withdrawal.
Stephenson et al. (2023) [81]	Australia	Case Study	To analyze the relationship between tapentadol prescription rates and deaths and review characteristics of tapentadol-related deaths.	A total of 12 cases studied where the cause of death was recorded as “tapentadol toxicity” or “mixed drug toxicity”.	≥15	Tapentadol; oxycodone; codeine; tramadol; hydromorphone; morphine.	Not stated	Not stated	Abuse; ADR; death; misuse; medication errors.
Gustafsson et al. (2023) [28]	Netherlands	Case study	To characterize the safety profile of opioids by conducting a descriptive study based on a spontaneous reporting system for ADRs in the Netherlands,	A total of 8769 ADR reports, including 112 reports of misuse and abuse and 21 of medication errors.	All ages	All opioids.	Not stated	Not stated	Abuse; ADR; medication errors; misuse.
Tardelli et al. (2023) [82]	USA	Cohort	To assess the effect of prescription amphetamines on retention in buprenorphine treatment and the risk of substance use disorder-related emergency admissions or drug-related poisonings.	A total of 90,269 patients with opioid use disorder, with 26,322 who had emergency admission and 8170 with drug-related poisoning.	12–64	Buprenorphine.	Yes	Not stated	Abuse; medication errors; misuse.
Lorenzini et al. (2022) [65]	Switzerland	Cohort	To evaluate the frequency of opioid-associated ADRs in emergency department visits.	A total of 64 patients visited the ED due to an opioid-related ADR. Among these, 16 overdoses were identified and 19 cases of abuse or misuse of opioids were identified.	Adults ≥18	Tramadol; morphine; buprenorphine; combinations of acetaminophen and naloxone.	Not stated	Not stated	ADRs; overdose; misuse; abuse; withdrawal.
Chiappini et al. (2022) [57]	Europe, USA	Case Study	To analyze ADRs from two major databases to detect abuse, misuse, dependence, and withdrawal issues related to selected opioids.	A total of 16,506 ADRs from EudraVigilance and 130,293 ADRs from FAERS.	All ages	All opioids.	Not stated	Not stated	Abuse; misuse; ADRs.
Schutijser et al. (2020) [58]	Netherlands	Cohort	To provide a detailed description of the underlying nature of opioid-related AEs.	From 10,917 patient records, 357 AEs were identified, of which 28 (8%) involved opioids.	All ages.	Fentanyl; tramadol; oxycodone; morphine; methadone.	Yes	Not stated	Medication errors.
Andreaggi et al. (2020) [80]	USA	Case study	To highlight the safety concerns surrounding opioids in clinical practice that were reported to a large post-marketing surveillance database, enhancing the understanding of opioid-related ADRs.	A total of 300,985 ADR with opioids were analyzed, with 22,167 (7.4%) cases of drug abuse and 11,454 (3.4%) of medication errors being identified.	Adults ≥18	Other: not specified.	Yes	Not stated	Abuse; medication errors.
Schifano et al. (2019) [84]	Worldwide: USA, Estonia, UK, France, Germany	Case Study	To analyze fentanyl misuse-, abuse-, dependence-, and withdrawal-related AEs identified within the EMA, YCS, and FAERS databases.	A total of 6151 individual cases reported to EMA, 1165 reported to YCS, and 19,145 reported to FAERS.	Adults ≥18	Fentanyl.	Yes	Yes	Abuse; misuse; dependence; withdrawal.
Mullan. et al. (2019) [54]	Australia	Case study	To compare rates and trends in hospital admissions due to medication misadventures for older adults with and without dementia.	A total of 131 cases of opioids and related analgesics causing adverse effects in therapeutic use in patients without dementia. A total of 906 opioids and related analgesics causing adverse effects in therapeutic use in patients with dementia.	≥65	Other: not specified.	Yes	Not stated	ADRs; medication errors.
Gariel et al. (2018) [49]	France	Case study	To determine the incidence of medication errors in pediatric anesthesia and identify characteristics and predictive factors.	A total of 10 cases of opioids involved in medication errors.	Pediatric ≤18	Alfentanil; remifentanil; sufentanil; morphine.	Yes	Not stated	Medication errors.
Eluri et al. (2018) [59]	USA	Cohort	To investigate characteristics and trends of analgesic medication errors reported to NPDS from 2000 through 2012.	A total of 123,613 reports, with 4093 health care facility admissions due to medication errors and 4616 serious outcomes of the medication error.	All ages	Hydrocodone; oxycodone; methadone; other: not specified.	Yes	No	Medication errors; misuse.
Moulis et al. (2018) [50]	France	Case study	To assess adverse ADRs due to medication errors with pediatric tramadol oral solution in France.	A total of 74 reports of serious ADRs were identified; among these, 13 were medication errors.	Pediatric ≤18	Tramadol.	Yes	Not stated	Medication errors.
Min et al. (2018) [60]	USA	Case study	To conduct an exploratory analysis on the demographics of those who reported painkiller-related AEs, examine the AEs most commonly associated with different types of painkillers, and identify potential safety signals.	A total of 18,696 AEs were associated with the use of opioids, with 2376 cases of abuse.	All ages	Other: not specified.	Yes	Not stated	Abuse.
Heneka et al. (2018) [73]	Australia	Cohort	To characterize and quantify opioid errors in palliative care services.	A total of 55 opioid errors were identified.	Adults ≥18	Morphine; hydromorphone; oxycodone; fentanyl.	Yes	Not stated	Medication errors.
Heneka et al. (2018) [74]	Australia	Case study	To identify and explore factors contributing to opioid medication errors in palliative care services.	A total of 78 opioid errors reported.	Adults ≥18	Hydromorphone; morphine; fentanyl; oxycodone; methadone; oxycodone/naloxone.	Yes	Not stated	Medication errors.
Seth et al. (2018) [79]	USA	Case study	To analyze drug overdose deaths, focusing on opioids, cocaine, and psychostimulants.	A total of 42,249 opioids overdose deaths in 2016.	All ages	Morphine; codeine; oxycodone; hydrocodone; heroin; methadone; fentanyl.	Yes	Yes	Abuse; death; overdose.
Day et al. (2016) [48]	USA	Case study	To compare the demographics, adverse events, and medical outcomes of patients who had unintentional hydrocodone or codeine exposures through the use of a state’s poison center database.	A total of 4607 codeine and 16,239 hydrocodone cases of unintentional exposure.	All ages	Codeine; hydrocodone.	Yes	Not stated	Medication errors; unintentional misuse.
Beaudoin et al. (2015) [75]	USA	Case study	To describe characteristics of patients with in-emergency department opioid-related adverse drug events, identify factors contributing to these events, and propose prevention strategies.	A total of 43 patients had a medication error resulting in harm.	≥40	Hydromorphone; morphine; fentanyl; multiple opioids.	Yes	Not stated	Medication errors.
Lovegrove et al. (2015) [69]	USA	Case study	To assess trends in ED visits for unsupervised medication exposures among children aged < 6 years from 2004 to 2013.	Opioids were the most commonly implicated medication class in ED visits involving prescription solid exposures, estimated at 4661 visits/year.	Pediatric ≤6	Tramadol; oxycodone; buprenorphine; other: hydrocodone.	Not stated	Not stated	Medication errors.
Brophy et al. (2014) [62]	USA	Case study	To characterize the trends of medication errors, including the age-related risks and the medications involved.	Opioids accounted for 54.9% (*n* = 4616) of all injuries due to analgesics, and of all opioid medication error calls, 3.7% resulted in an injury. For adults aged 20–49 years, opioids were the leading class of analgesics involved in medication errors (*n* = 43,869, 36.7%).	All ages	Hydrocodone.	Yes	Yes	Medication errors.
Lövborg et al. (2014) [63]	Sweden	Case study	To describe and characterize medication errors regarding transdermal opioid patches containing fentanyl and buprenorphine submitted to a regional incident reporting system.	A total of 151 MEs were identified as related to transdermal patches containing fentanyl or buprenorphine. Common errors were wrong administration time, with 67 instances (44%), wrong dose, with 34 instances (23%), and omission of dose, with 20 instances (13%). Of all MEs, 118 (78%) occurred in the administration stage of the medication process. Harm was reported in 26 (17%) of the included cases, of which 2 cases caused serious harm (nausea/vomiting and respiratory depression).	Not specified	Fentanyl; buprenorphine.	Yes	Not stated	Medication errors.
McDonnell et al. (2011) [51]	Canada	Case study	To review and characterize pediatric opioid medication errors reported at The Hospital for Sick Children.	A total of 507 medication-related safety reports involving opioids.	Pediatric ≤18	Morphine; fentanyl; hydromorphone; codeine; oxycodone.	Yes	Yes	Medication errors.
Cassidy et al. (2011) [55]	Ireland	Case study	To characterize the epidemiology and type of medication errors reported to the National Poisons Information Centre of Ireland.	A total of 52 cases of opioid analgesics and 17 cases of paracetamol+codeine involved in medication errors.	All ages	Other: not specified; codeine.	Yes	Not stated	Medication errors.
Culleré et al. (2009) [67]	Spain	Cohort	To determine the incidence rate of AEs in hospitalized patients and evaluate the event prevention percentage.	A total of 18 cases of preventable errors associated with opiates.	Adults ≥18	Other: not specified.	Yes	Not stated	Medication errors.
Bailey et al. (2009) [68]	USA	Case study	To test the hypothesis that prescription opioid poisoning of young children occurs regularly and is associated with serious health events, including death.	A total of 9179 children under 6 years old were exposed to opioids. A total of 51 patients had a major effect (*n* = 43) or death (*n* = 8) as the outcome. In addition, 214 moderate effects were reported.	Pediatric ≤6	Fentanyl; oxycodone; morphine; buprenorphine; other: hydrocodone; hydromorphone; methadone.	Yes	Not stated	Death; medication errors.
Boockvar et al. (2009) [66]	USA	Cohort	To quantify the rate of AEs caused by prescribing discrepancies and the discrimination of an index of high-risk transition drug prescribing.	A total of 54 patients had opioid prescription discrepancies, and 15 of these caused an AE, being the drug class that caused the most AEs.	Not specified	Other: not specified.	Yes	No	Medication errors.
Cobaugh et al. (2006) [53]	Not stated	Case study	To characterize the severity of hazards posed by medications implicated in the poisoning of older adults. Hazard factors were calculated from data derived from medication-related calls to poison centers due to ADRs and therapeutic errors in patients 60 years of age or older.	A total of 543 cases related to exposure to therapeutic errors using opioids.	≥60	Morphine; other: not specified.	Yes	No	Medication errors.
Hicks et al. (2006) [52]	USA	Case study	To identify recurring products and therapeutic classes most commonly associated with reported pediatric medication errors.	A total of 96 (11.5%) reports of harmful errors involving opioid analgesics.	Pediatric ≤17	Fentanyl; morphine.	Yes	No	Medication errors.
Whipple et al. (1992) [64]	USA	Cohort	To determine the causes and frequency of overdoses associated with the administration of opioid analgesics in hospitalized patients.	A total of 81 overdoses caused by opioids, from which 22 were preventable.	Not specified	Morphine, fentanyl; meperidine; levodromoran.	Yes	No	Medication errors; overdose.

ADR: adverse drug reaction; AEs: adverse events; ED: emergency department; EMA: European Medicines Agency; FAERS: FDA Adverse Event Reporting System; YCS: Yellow Card Scheme (a UK system for collecting information on suspected adverse drug reactions); “Diversion or recreational use” refers to the practice of redirecting prescribed opioids from their intended medical purpose to unauthorized or illicit use; Table 1 summarizes the key findings from the included studies examining opioid-related medication errors, abuse, and AEs. These studies cover multiple countries and involve different populations characteristics, providing a comprehensive overview of the issues associated with opioid use. The table shows information on the country of the study, the study design, the purpose of the study, the number of reports or patients involved, the age group, the opioids used, the type of use, distinguishing the use as prescribed medicines or diversion use, and the type of problems reported such as abuse, misuse, AEs, and medication errors.

## Data Availability

Requests to access the datasets should be directed to the corresponding author and will be granted upon reasonable request.

## Supplementary Materials

The following supporting information can be downloaded at: https://www.mdpi.com/article/10.3390/ph17081009/s1, Table S1: PRISMA 2020 Checklist; Table S2: Search strategy for PubMed database; Table S3: Search strategy for Scopus database; Table S4: Search strategy for EBSCO database; Table S5: Data extraction template; Table S6: Risk of bias evaluation of the cohort studies included in this study; Table S7: Risk of bias evaluation of the case studies included in this study.

## References

[1] Ferner R.E., McGettigan P. (2018). Adverse drug reactions. BMJ.

[2] Callahan D. (1998). Managed care and the goals of medicine. J. Am. Geriatr. Soc..

[3] Coleman J.J., Pontefract S.K. (2016). Adverse drug reactions. Clin. Med..

[4] Jay G.W., Heit H.A., Gourlay D.L. (2019). When the best of intentions leads to bad outcomes. Pain Ther..

[5] Bouvy J.C., De Bruin M.L., Koopmanschap M.A. (2015). Epidemiology of adverse drug reactions in Europe: A review of recent observational studies. Drug Saf..

[6] Rashed A.N., Wong I.C., Cranswick N., Hefele B., Tomlin S., Jackman J., Lee K., Hon K.L., Ong J., Ghaleb M. (2012). Adverse Drug Reactions in Children—International Surveillance and Evaluation (ADVISE) A Multicentre Cohort Study. Drug Saf..

[7] van Hunsel F., Ekhart C. (2021). Unexpected beneficial effects of drugs: An analysis of cases in the Dutch spontaneous reporting system. Eur. J. Clin. Pharmacol..

[8] Edwards I.R., Aronson J.K. (2000). Adverse drug reactions: Definitions, diagnosis, and management. Lancet.

[9] Laurence D.R., Carpenter J. (1998). A Dictionary of Pharmacology and Allied Topics.

[10] World Health Organization (1972). International drug monitoring: The role of national centres. Proceedings of the Report of a WHO Meeting.

[11] Parliament E., The Council of the European Union (2010). Directive 2010/84/EU of the European Parliament and of the Council of 15 December 2010 amending, as regards pharmacovigilance, Directive 2001/83/EC on the Community code relating to medicinal products for human use. OJ.

[12] Agency E.M. (2014). Guideline on Good Pharmacovigilance Practices (GVP), Module VI—Management and Reporting of Adverse Reactions to Medicinal Products, 1st Revised.

[13] Baldo P., Francescon S., Fornasier G. (2018). Pharmacovigilance workflow in Europe and Italy and pharmacovigilance terminology. Int. J. Clin. Pharm..

[14] Morimoto T., Gandhi T.K., Seger A.C., Hsieh T.C., Bates D.W. (2004). Adverse drug events and medication errors: Detection and classification methods. Qual. Saf. Health Care.

[15] Aronson J.K. (2009). Medication errors: What they are, how they happen, and how to avoid them. QJM.

[16] Ferner R.E., Aronson J.K. (2006). Clarification of terminology in medication errors: Definitions and classification. Drug Saf..

[17] Gandhi T.K., Seger D.L., Bates D.W. (2000). Identifying drug safety issues: From research to practice. Int. J. Qual. Health Care.

[18] Ackroyd-Stolarz S., Hartnell N., Mackinnon N.J. (2006). Demystifying medication safety: Making sense of the terminology. Res. Soc. Adm. Pharm..

[19] Pathan H., Williams J. (2012). Basic opioid pharmacology: An update. Br. J. Pain.

[20] Fields H.L. (2011). The doctor’s dilemma: Opiate analgesics and chronic pain. Neuron.

[21] Stein C. (2016). Opioid receptors. Annu. Rev. Med..

[22] Waldhoer M., Bartlett S.E., Whistler J.L. (2004). Opioid receptors. Annu. Rev. Biochem..

[23] Branford R., Droney J., Ross J. (2012). Opioid genetics: The key to personalized pain control?. Clin. Genet..

[24] Smith M., Kong D., Kuo A., Imam M., Williams C. (2022). Analgesic opioid ligand discovery based on nonmorphinan scaffolds derived from natural sources. J. Med. Chem..

[25] Vijayvargiya P., Camilleri M., Vijayvargiya P., Erwin P., Murad M. (2020). Systematic review with meta-analysis: Efficacy and safety of treatments for opioid-induced constipation. Aliment. Pharmacol. Ther..

[26] Polati E., Nizzero M., Rama J., Martini A., Gottin L., Donadello K., Del Balzo G., Varrassi G., Marinangeli F., Vittori A. (2022). Oxycodone-naloxone combination hinders opioid consumption in osteoarthritic chronic low back pain: A retrospective study with two years of follow-up. Int. J. Environ. Res. Public Health.

[27] Ahlbeck K. (2011). Opioids: A two-faced janus. Curr. Med. Res. Opin..

[28] Gustafsson M., Matos C., Joaquim J., Scholl J., van Hunsel F. (2023). Adverse drug reactions to opioids: A study in a national pharmacovigilance database. Drug Saf..

[29] Daoust R., Paquet J., Lavigne G., Piette É., Chauny J. (2015). Impact of age, sex and route of administration on adverse events after opioid treatment in the emergency department: A retrospective study. Pain Res. Manag..

[30] Volkow N., Koroshetz W. (2017). Lack of evidence for benefit from long-term use of opioid analgesics for patients with neuropathy. JAMA Neurol..

[31] Macey T., Bobeck E., Hegarty D., Aicher S., Ingram S., Morgan M. (2009). Extracellular signal-regulated kinase 1/2 activation counteracts morphine tolerance in the periaqueductal gray of the rat. J. Pharmacol. Exp. Ther..

[32] Bialas P., Maier C., Klose P., Häuser W. (2020). Efficacy and harms of long-term opioid therapy in chronic non-cancer pain: Systematic review and meta-analysis of open-label extension trials with a study duration ≥ 26 weeks. Eur. J. Pain.

[33] Maier C., Schaub C., Willweber-Strumpf A., Zenz M. (2005). Long-term efficiency of opioid medication in patients with chronic non-cancer-associated pain: Results of a survey 5 years after onset of medical treatment. Der. Schmerz..

[34] Hoffman E., Watson J., Sauver J., Staff N., Klein C. (2017). Association of long-term opioid therapy with functional status, adverse outcomes, and mortality among patients with polyneuropathy. JAMA Neurol..

[35] Ni J., Tang X., Chen L. (2023). Medication overdose data analysis: A review of medication error reports in the FDA adverse event reporting system (FAERS). BMC Pharmacol. Toxicol..

[36] Hedegaard H., Miniño A.M., Warner M. (2020). Drug Overdose Deaths in the United States, 1999–2018.

[37] Compton W.M., Jones C.M. (2019). Epidemiology of the U.S. opioid crisis: The importance of the vector. Ann. N. Y. Acad. Sci..

[38] Cranston K., Alpren C., John B., Dawson E., Roosevelt K., Burrage A., Bryant J., Switzer W.M., Breen C., Peters P.J. (2019). Notes from the field: HIV diagnoses among persons who inject drugs—Northeastern Massachusetts, 2015–2018. Morb. Mortal. Wkly. Rep..

[39] Jones C., Einstein E., Compton W. (2018). Changes in synthetic opioid involvement in drug overdose deaths in the united states, 2010–2016. JAMA.

[40] Compton W., Valentino R., DuPont R. (2020). Polysubstance use in the U.S. opioid crisis. Mol. Psychiatry.

[41] Health T.L. (2022). Opioid overdose crisis: Time for a radical rethink. Lancet Public Health.

[42] Mojtabai R., Amin-Esmaeili M., Nejat E., Olfson M. (2019). Misuse of prescribed opioids in the United States. Pharmacoepidemiol. Drug Saf..

[43] Kalkman G.A., van den Brink W., Pierce M., Atsma F., Vissers K.C.P., Schers H.J., van Dongen R.T.M., Kramers C., Schellekens A.F.A. (2022). Monitoring opioids in Europe: The need for shared definitions and measuring drivers of opioid use and related harms. Eur. Addict. Res..

[44] Shei A., Hirst M., Kirson N., Enloe C., Birnbaum H., Dunlop W. (2015). Estimating the health care burden of prescription opioid abuse in five European countries. Clin. Outcomes Res..

[45] Page M.J., McKenzie J.E., Bossuyt P.M., Boutron I., Hoffmann T.C., Mulrow C.D., Shamseer L., Tetzlaff J.M., Akl E.A., Brennan S.E. (2021). The PRISMA 2020 statement: An updated guideline for reporting systematic reviews. Int. J. Surg..

[46] Kellermeyer L., Harnke B., Knight S. (2018). Covidence and rayyan. J. Med. Libr. Assoc..

[47] Marraffa J.M., Stork C.M., Hoffman R.S., Su M.K. (2018). Poison control center experience with tianeptine: An unregulated pharmaceutical product with potential for abuse. Clin. Toxicol..

[48] Day L., Kleinschmidt K., Forrester M.B., Feng S.-Y. (2016). Comparison of unintentional exposures to codeine and hydrocodone reported to Texas poison centers. J. Emerg. Med..

[49] Gariel C., Cogniat B., Desgranges F.P., Chassard D., Bouvet L. (2018). Incidence, characteristics, and predictive factors for medication errors in paediatric anaesthesia: A prospective incident monitoring study. Br. J. Anaesth..

[50] Moulis F., Durrieu G., Masmoudi K., Boyer M.G., Rocher F., Montastruc F., Montastruc J.-L. (2018). Medication errors with tramadol drops in children. Eur. J. Clin. Pharmacol..

[51] Mc Donnell C. (2011). Opioid medication errors in pediatric practice: Four years’ experience of voluntary safety reporting. Pain Res. Manag..

[52] Hicks R.W., Becker S.C., Cousins D.D. (2006). Harmful medication errors in children: A 5-year analysis of data from the USP’s MEDMARX^®^program. J. Pediatr. Nurs..

[53] Cobaugh D.J., Krenzelok E.P. (2006). Adverse drug reactions and therapeutic errors in older adults: A hazard factor analysis of poison center data. Am. J. Health Pharm..

[54] Mullan J., Burns P., Mohanan L., Lago L., Jordan M., Potter J. (2019). Hospitalisation for medication misadventures among older adults with and without dementia: A 5-year retrospective study. Australas. J. Ageing.

[55] Cassidy N., Duggan E., Williams D.J.P., Tracey J.A. (2011). The epidemiology and type of medication errors reported to the National Poisons Information Centre of Ireland. Clin. Toxicol..

[56] Jobski K., Bantel C., Hoffmann F. (2023). Characteristics and completeness of spontaneous reports by reporter’s role in Germany: An analysis of the EudraVigilance database using the example of opioid-associated abuse, dependence, or withdrawal. Pharmacol. Res. Perspect..

[57] Chiappini S., Vickers-Smith R., Guirguis A., Corkery J.M., Martinotti G., Harris D.R., Schifano F. (2022). Pharmacovigilance signals of the opioid epidemic over 10 years: Data mining methods in the analysis of pharmacovigilance datasets collecting adverse drug reactions (ADRs) Reported to EudraVigilance (EV) and the FDA Adverse Event Reporting System (FAERS). Pharmaceuticals.

[58] Schutijser B.C.F.M., Jongerden I., Klopotowska J.E., Moesker M., Langelaan M., Wagner C., de Bruijne M. (2020). Nature of adverse events with opioids in hospitalised patients: A post-hoc analysis of three patient record review studies. BMJ Open.

[59] Eluri M., A Spiller H., Casavant M.J., Chounthirath T., A Conner K., A Smith G. (2018). Analgesic-related medication errors reported to US poison control centers. Pain Med..

[60] Min J., Osborne V., Kowalski A., Prosperi M. (2018). Reported adverse events with painkillers: Data mining of the US Food and Drug Administration adverse events reporting system. Drug Saf..

[61] Madeiro A.C., Dayana P., Carrilho L., Bonfim M., Braqueais A.R., Elisângela F., Lima T. (2010). Adesão de portadores de insuficiência renal crônica ao tratamento de hemodiálise. Acta Paul. Enferm..

[62] Brophy T.J., Spiller H.A., Casavant M.J., Chounthirath T., Smith M.D., Xiang H. (2014). Medication errors reported to US poison control centers, 2000–2012. Clin. Toxicol..

[63] Lövborg H., Holmlund M., Hägg S. (2014). Medication errors related to transdermal opioid patches: Lessons from a regional incident reporting system. BMC Pharmacol. Toxicol..

[64] Whipple J.K., Ausman R.K., Quebbeman E.J. (1992). Narcotic Use in the Hospital: Reasonably Safe?.

[65] Ing Lorenzini K., Wainstein L., Spechbach H., Sarasin F., Ramlawi M., Desmeules J., Piguet V. (2022). Opioid-related adverse drug reactions in patients visiting the emergency division of a tertiary hospital. Pharmacol. Res. Perspect..

[66] Boockvar K.S., Liu S., Goldstein N., Nebeker J., Siu A., Fried T. (2009). Prescribing discrepancies likely to cause adverse drug events after patient transfer. BMJ Qual. Saf..

[67] Culleré C.B., Torner M.G., Ruiz J.A., Creus M.T., Martín M.B., Sunyer M.C., Gubert M.T., Cortinas M.C., Sabaté E.B., Solà J.F. (2009). Detecting adverse drug events during the hospital stay. Farm. Hosp..

[68] Bailey J.E., Campagna E., Dart R.C. (2009). The underrecognized toll of prescription opioid abuse on young children. Ann. Emerg. Med..

[69] Lovegrove M.C., Weidle N.J., Budnitz D.S. (2015). Trends in emergency department visits for unsupervised pediatric medication exposures, 2004–2013. Pediatrics.

[70] McMaughan D.J., Oloruntoba O., Smith M.L. (2020). Socioeconomic status and access to healthcare: Interrelated drivers for healthy aging. Front. Public Health.

[71] Jones T.A., Como J.A. (2003). Assessment of medication errors that involved drug allergies at a university hospital. Pharmacother. J. Hum. Pharmacol. Drug Ther..

[72] Ni Y., Lingren T., Huth H., Timmons K., Melton K., Kirkendall E.S. (2020). Integrating and evaluating the data quality and utility of smart pump information in detecting medication administration errors: Evaluation study. JMIR Med. Inform..

[73] Heneka N., Shaw T., Rowett D., Lapkin S., Phillips J.L. (2018). Opioid errors in inpatient palliative care services: A retrospective review. BMJ Support. Palliat. Care.

[74] Heneka N., Shaw T., Rowett D., Lapkin S., Phillips J.L. (2018). Exploring factors contributing to medication errors with opioids in Australian specialist palliative care inpatient services: A multi-incident analysis. J. Palliat. Med..

[75] Beaudoin F.L., Merchant R.C., Janicki A., McKaig D.M., Babu K.M. (2015). Preventing iatrogenic overdose: A review of in–emergency department opioid-related adverse drug events and medication errors. Ann. Emerg. Med..

[76] McDonald H.P., Garg A.H.R. (2002). Interventions to enhance patient adherence to medication prescriptions: Scientific review. J. Am. Med. Assoc..

[77] Harris E., Harms M., Cao D., Prestwood C., DeBinya L., Kleinschmidt K., Young A., Saha S., Rule J., Alvarez K. (2023). Declining rates of opioid/acetaminophen combination product overdose: 2011–2020. Hepatol. Commun..

[78] France H.S., Aronson J.K., Heneghan C., Ferner R.E., Cox A.R., Richards G.C. (2023). Preventable deaths involving medicines: A systematic case series of coroners’ reports 2013–2022. Drug Saf..

[79] Seth P. (2018). Overdose deaths involving opioids, cocaine, and psychostimulants—United States, 2015–2016. MMWR Morb. Mortal. Wkly. Rep..

[80] Andreaggi C.A., Novak E.A., Mirabile M.E., Sampathkumar S., Gray M.P., He M., Kane-Gill S.L. (2020). Safety concerns reported by consumers, manufacturers and healthcare professionals: A detailed evaluation of opioid-related adverse drug reactions in the FDA database over 15 years. Pharmacoepidemiol. Drug Saf..

[81] Stephenson L., van den Heuvel C., Humphries M., Scott T., Byard R.W. (2024). Increased incidence of mixed drug toxicity deaths involving tapentadol–A forensic study. Med. Sci. Law.

[82] Tardelli V., Xu K.Y., Bisaga A., Levin F.R., Fidalgo T.M., Grucza R.A. (2023). Prescription amphetamines in people with opioid use disorder and co-occurring psychostimulant use disorder initiating buprenorphine: An analysis of treatment retention and overdose risk. BMJ Ment. Health.

[83] Chatterton C.N., Handy R.P. (2023). Fentanyl concentrations in ligated femoral blood in the presence and absence of NPS benzodiazepine drugs. A review of over 1250 benzo-dope/fentanyl toxicity cases in Alberta Canada. Forensic Sci. Int..

[84] Schifano F., Chiappini S., Corkery J.M., Guirguis A. (2019). Assessing the 2004–2018 fentanyl misusing issues reported to an international range of adverse reporting systems. Front. Pharmacol..

[85] Dunbar-Jacob J., Mortimer-Stephens M.K. (2001). Treatment adherence in chronic disease. J. Clin. Epidemiol..

[86] Chou R., Turner J.A., Devine E.B., Hansen R.N., Sullivan S.D., Blazina I., Dana T., Bougatsos C., Deyo R.A. (2015). The effectiveness and risks of long-term opioid therapy for chronic pain: A systematic review for a National Institutes of Health Pathways to Prevention Workshop. Ann. Intern. Med..

[87] Kyung E.J., Ryu J.H., Kim E.Y. (2013). Evaluation of adverse reactions to contrast media in the hospital. Br. J. Radiol..

[88] Trojan A., Beil H.W. (1978). Tilidine abuse and dependence. Drug Alcohol. Depend..

[89] Radbruch L., Glaeske G., Grond S., Münchberg F., Scherbaum N., Storz E., Tholen K., Zagermann-Muncke P., Zieglgänsberger W., Hoffmann-Menzel H. (2013). Topical review on the abuse and misuse potential of tramadol and tilidine in Germany. Subst. Abus..

[90] Vlaams Expertisecentrum Alcohol en Andere Drugs (2018). Dossier Opioïde Pijnstillers. https://www.vad.be/catalogus/detail/dossier-opioide-pijnstillers.

[91] Drewes A.M., Jensen R.D., Nielsen L.M., Droney J., Christrup L.L., Arendt-Nielsen L., Riley J., Dahan A. (2013). Differences between opioids: Pharmacological, experimental, clinical and economical perspectives. Br. J. Clin. Pharmacol..

[92] Wightman R., Perrone J., Portelli I., Nelson L. (2012). Likeability and abuse liability of commonly prescribed opioids. J. Med. Toxicol..

[93] Cicero T.J., Ellis M.S., Surratt H.L., Kurtz S.P. (2014). The changing face of heroin use in the United States: A retrospective analysis of the past 50 years. JAMA Psychiatry.

[94] Lynn E., Cousins G., Lyons S., Bennett K.E. (2021). Trends in drug poisoning deaths, by sex, in Ireland: A repeated cross-sectional study from 2004 to 2017. BMJ Open.

[95] Food and Drug Administration (2024). Timeline of Selected FDA Activities and Significant Events Addressing Substance Use and Overdose Prevention. https://www.fda.gov/drugs/food-and-drug-administration-overdose-prevention-framework/timeline-selected-fda-activities-and-significant-events-addressing-substance-use-and-overdose.

[96] Ferner R.E., Easton C., Cox A.R. (2018). Deaths from medicines: A systematic analysis of coroners’ reports to prevent future deaths. Drug Saf..

[97] Booth S., Gloag M., Kinna S., Bell A., Wheble J., Wheeler D. (2015). Pictorial prescribing reduces fentanyl drug administration errors: A simulated controlled study. BMJ Support. Palliat. Care.

[98] Vadivelu N., Schermer E., Kodumudi G., Berger J. (2016). The clinical applications of extended-release abuse-deterrent opioids. CNS Drugs.

[99] Ratycz M.C., Papadimos T.J., Vanderbilt A.A. (2018). Addressing the growing opioid and heroin abuse epidemic: A call for medical school curricula. Med. Educ. Online.

[100] Ostling P.S., Davidson K.S., Anyama B.O., Helander E.M., Wyche M.Q., Kaye A.D. (2018). America’s opioid epidemic: A comprehensive review and look into the rising crisis. Curr. Pain Headache Rep..

[101] Manchikanti L., Sanapati J., Benyamin R.M., Atluri S., Kaye A.D., Hirsch J.A. (2018). Reframing the prevention strategies of the opioid crisis: Focusing on prescription opioids, fentanyl, and heroin epidemic. Pain Physician.

[102] Sabblah G.T., Seaneke S.K., Kushitor M., van Hunsel F., Taxis K., Duwiejua M., van Puijenbroek E. (2022). Evaluation of pharmacovigilance systems for reporting medication errors in Africa and the role of patients using a mixed-methods approach. PLoS ONE.

[103] Reinhart M., Scarpati L., Kirson N., Patton C., Shak N., Erensen J. (2018). The economic burden of abuse of prescription opioids: A systematic literature review from 2012 to 2017. Appl. Health Econ. Health Policy.

[104] Florence C.S., Zhou C., Luo F., Xu L. (2016). The economic burden of prescription opioid overdose, abuse, and dependence in the United States, 2013. Med. Care.

[105] Kolodny A. (2020). How FDA failures contributed to the opioid crisis. AMA J. Ethics.

[106] Yakubi H., Gac B., Apollonio D.E. (2022). Industry strategies to market opioids to children and women in the USA: A content analysis of internal industry documents from 1999 to 2017 released in State of Oklahoma v. Purdue Pharma, LP et al. BMJ Open.

[107] Feldscher K. (2022). What Led to the Opioid Crisis—And How to Fix It. Harvard Sch. Public Health.

[108] Jantarada C., Silva C., Guimarães-Pereira L. (2021). Prevalence of Problematic Use of Opioids in Patients with Chronic Noncancer Pain: A Systematic Review with Meta-analysis. Pain Pract..

[109] Vowles K.E., McEntee M.L., Julnes P.S., Frohe T., Ney J.P., Van Der Goes D.N. (2015). Rates of opioid misuse, abuse, and addiction in chronic pain: A systematic review and data synthesis. Pain.

[110] Phillips J.K., Ford M.A., Bonnie R.J. (2017). National Academies of Sciences Engineering, and Medicine; Health and Medicine Division; Board on Health Sciences Policy; Committee on Pain Management and Regulatory Strategies to Address Prescription Opioid Abuse. Pain Management and the Opioid Epidemic: Balancing Societal and Individual Benefits and Risks of Prescription Opioid Use.

[111] Matos C., Rodrigues L., Joaquim J. (2017). Attitudes and opinions of Portuguese community pharmacy professionals towards patient reporting of adverse drug reactions and the pharmacovigilance system. Drugs Ther. Perspect..

[112] van Hoof M., Chinchilla K., Härmark L., Matos C., Inácio P., van Hunsel F. (2022). Factors Contributing to Best Practices for Patient Involvement in Pharmacovigilance in Europe: A Stakeholder Analysis. Drug Saf..

[113] Virbalas J., Morrow B.E., Reynolds D., Bent J.P., Ow T.J. (2019). The prevalence of Ultrarapid metabolizers of Codeine in a diverse urban population. Otolaryngol. Neck Surg..

[114] Adler N.E., Boyce T., Chesney M.A., Cohen S., Folkman S., Kahn R.L., Syme S.L. (1994). Socioeconomic status and health: The challenge of the gradient. Am. Psychol..

[115] van Stekelenborg J., Kara V., Haack R., Vogel U., Garg A., Krupp M., Gofman K., Dreyfus B., Hauben M., Bate A. (2023). Individual case safety report replication: An analysis of case reporting transmission networks. Drug Saf..

[116] Lopez-Gonzalez E., Herdeiro M.T., Figueiras A. (2009). Determinants of under-reporting of adverse drug reactions: A systematic review. Drug Saf..

[117] Varallo F.R., Guimarães S.D.O.P., Abjaude S.A.R., Mastroianni P.D.C. (2014). Causes for the underreporting of adverse drug events by health professionals: A systematic review. Rev. Esc. Enferm. USP.

[118] Härmark L., Van Hunsel F., Grundmark B. (2015). ADR reporting by the general public: Lessons learnt from the Dutch and Swedish systems. Drug Saf..

[119] Moore M.D., Ali S., Burnich-Line D., Gonzales W., Stanton M.V. (2020). Stigma Opioids, and Public Health Messaging: The Need to Disentangle Behavior From Identity. Am. J. Public Health.

