# Supplementary material for: Misuse, Abuse and Medication Errors’ Adverse Events Associated with Opioids—A Systematic Review
Source: Pharmaceuticals (Basel). 2024 Jul 31;17(8):1009. doi: 10.3390/ph17081009 (PMC11357286; doi:10.3390/ph17081009)
Supplement: Supplementary file 1 [file pharmaceuticals-17-01009-s001.zip › pharmaceuticals-3119774-supplementary.pdf]

Review

Misuse, Abuse and Medication Errors' Adverse Events Associated with Opioids—A Systematic Review

Moa Gustafsson [1], Vítor Silva [2], Carolina Valeiro [3], João Joaquim [4], Florence van Hunsel [5,6] and Cristiano Matos [4,7,*]

[1] Department of Pharmacology, Institute of Neuroscience and Physiology, The Sahlgrenska Academy, University of Gothenburg, 41390 Gothenburg, Sweden; moa.gustafsson98@icloud.com
[2] Unidade Local de Saúde de Coimbra, EPE, 3004-561 Coimbra, Portugal; vahsilva@hotmail.com
[3] Eu2P European Programme in Pharmacovigilance and Pharmacoepidemiology, University Autónoma de Barcelona, 08193 Barcelona, Spain; carolinavaleiro99@gmail.com
[4] Instituto Politécnico De Coimbra, ESTESC-Coimbra Health School, Farmácia, 3046-854 Coimbra, Portugal; jjj@estesc.ipc.pt
[5] Netherlands Pharmacovigilance Centre Lareb, 5237 MH 's-Hertogenbosch, The Netherlands; f.vanhunsel@lareb.nl
[6] Department of PharmacoTherapy, Epidemiology & Economics, Groningen Research Institute of Pharmacy (GRIP), University of Groningen, 9747 AG Groningen, The Netherlands
[7] QLV Research Consulting, 3030-193 Coimbra, Portugal
* Correspondence: cristiano.r.matos@gmail.com

Supplementary Materials: The following supporting information is part of the paper "Misuse, Abuse and Medication Errors' Adverse Events Associated with Opioids—A Systematic Review" from Gustafsson et al. (2024)

Table S1. PRISMA 2020 Checklist.

Table S2. Search strategy for PubMed database

Table S3. Search strategy for Scopus database

Table S4. Search strategy for EBSCO database

Table S5. Data extraction template

Table S6. Risk of bias evaluation of the cohort studies included in this study.

Table S7. Risk of bias evaluation of the case studies included in this study.

Table S1. PRISMA 2020 Checklist

PRISMA 2020 Checklist

Section and Topic	Item #	Checklist item	Indicate if the item is reported
**TITLE**			
Title	1	Identify the report as a systematic review.	**Yes**
**ABSTRACT**			
Abstract	2	See the PRISMA 2020 for Abstracts checklist.	**Yes**
**INTRODUCTION**			
Rationale	3	Describe the rationale for the review in the context of existing knowledge.	**Yes**
Objectives	4	Provide an explicit statement of the objective(s) or question(s) the review addresses.	**Yes**
**METHODS**			
Eligibility criteria	5	Specify the inclusion and exclusion criteria for the review and how studies were grouped for the syntheses.	**Yes**
Information sources	6	Specify all databases, registers, websites, organisations, reference lists and other sources searched or consulted to identify studies. Specify the date when each source was last searched or consulted.	**Yes**
Search strategy	7	Present the full search strategies for all databases, registers and websites, including any filters and limits used.	**Yes**
Selection process	8	Specify the methods used to decide whether a study met the inclusion criteria of the review, including how many reviewers screened each record and each report retrieved, whether they worked independently, and if applicable, details of automation tools used in the process.	**Yes**
Data collection process	9	Specify the methods used to collect data from reports, including how many reviewers collected data from each report, whether they worked independently, any processes for obtaining or confirming data from study investigators, and if applicable, details of automation tools used in the process.	**Yes**
Data items	10a	List and define all outcomes for which data were sought. Specify whether all results that were compatible with each outcome domain in each study were sought (e.g. for all measures, time points, analyses), and if not, the methods used to decide which results to collect.	**Yes**
	10b	List and define all other variables for which data were sought (e.g. participant and intervention characteristics, funding sources). Describe any assumptions made about any missing or unclear information.	**Yes**
Study risk of bias assessment	11	Specify the methods used to assess risk of bias in the included studies, including details of the tool(s) used, how many reviewers assessed each study and whether they worked independently, and if applicable, details of automation tools used in the process.	**Yes**
Effect measures	12	Specify for each outcome the effect measure(s) (e.g. risk ratio, mean difference) used in the synthesis or presentation of results.	**Yes**
Synthesis methods	13a	Describe the processes used to decide which studies were eligible for each synthesis (e.g. tabulating the study intervention characteristics and comparing against the planned groups for each synthesis (item #5)).	**Yes**
	13b	Describe any methods required to prepare the data for presentation or synthesis, such as handling of missing summary statistics, or data conversions.	**Yes**
	13c	Describe any methods used to tabulate or visually display results of individual studies and syntheses.	**Yes**
	13d	Describe any methods used to synthesize results and provide a rationale for the choice(s). If meta-analysis was performed, describe the model(s), method(s) to identify the presence and extent of statistical heterogeneity, and software package(s) used.	**Yes**
	13e	Describe any methods used to explore possible causes of heterogeneity among study results (e.g. subgroup analysis, meta-regression).	**Yes**
	13f	Describe any sensitivity analyses conducted to assess robustness of the synthesized results.	**Yes**
Reporting bias assessment	14	Describe any methods used to assess risk of bias due to missing results in a synthesis (arising from reporting biases).	**Yes**
Certainty assessment	15	Describe any methods used to assess certainty (or confidence) in the body of evidence for an outcome.	**Yes**

PRISMA 2020 Checklist

Section and Topic	Item #	Checklist item	Indicate if the item is reported
**RESULTS**			
Study selection	16a	Describe the results of the search and selection process, from the number of records identified in the search to the number of studies included in the review, ideally using a flow diagram.	**Yes**
	16b	Cite studies that might appear to meet the inclusion criteria, but which were excluded, and explain why they were excluded.	**Yes**
Study characteristics	17	Cite each included study and present its characteristics.	**Yes**
Risk of bias in studies	18	Present assessments of risk of bias for each included study.	**Yes**
Results of individual studies	19	For all outcomes, present, for each study: (a) summary statistics for each group (where appropriate) and (b) an effect estimate and its precision (e.g. confidence/credible interval), ideally using structured tables or plots.	**Yes**
Results of syntheses	20a	For each synthesis, briefly summarise the characteristics and risk of bias among contributing studies.	**Yes**
	20b	Present results of all statistical syntheses conducted. If meta-analysis was done, present for each the summary estimate and its precision (e.g. confidence/credible interval) and measures of statistical heterogeneity. If comparing groups, describe the direction of the effect.	**Yes**
	20c	Present results of all investigations of possible causes of heterogeneity among study results.	**Yes**
	20d	Present results of all sensitivity analyses conducted to assess the robustness of the synthesized results.	**Yes**
Reporting biases	21	Present assessments of risk of bias due to missing results (arising from reporting biases) for each synthesis assessed.	**Yes**
Certainty of evidence	22	Present assessments of certainty (or confidence) in the body of evidence for each outcome assessed.	**Yes**
**DISCUSSION**			
Discussion	23a	Provide a general interpretation of the results in the context of other evidence.	**Yes**
	23b	Discuss any limitations of the evidence included in the review.	**Yes**
	23c	Discuss any limitations of the review processes used.	**Yes**
	23d	Discuss implications of the results for practice, policy, and future research.	**Yes**
**OTHER INFORMATION**			
Registration and protocol	24a	Provide registration information for the review, including register name and registration number, or state that the review was not registered.	**Yes**
	24b	Indicate where the review protocol can be accessed, or state that a protocol was not prepared.	**Yes**
	24c	Describe and explain any amendments to information provided at registration or in the protocol.	**Yes**
Support	25	Describe sources of financial or non-financial support for the review, and the role of the funders or sponsors in the review.	**Yes**
Competing interests	26	Declare any competing interests of review authors.	**Yes**
Availability of data, code and other materials	27	Report which of the following are publicly available and where they can be found: template data collection forms; data extracted from included studies; data used for all analyses; analytic code; any other materials used in the review.	**Yes**

From: Page MJ, McKenzie JE, Bossuyt PM, Boutron I, Hoffmann TC, Mulrow CD, et al. The PRISMA 2020 statement: an updated guideline for reporting systematic reviews. BMJ 2021;372:n71. doi: 10.1136/bmj.n71

Table S2. Search strategy for PubMed database

Search #	Query	Items found
# 01	**Search: (medication error OR abuse OR misuse)**<br>medication error: "medication errors"[MeSH Terms] OR ("medication"[All Fields] AND "errors"[All Fields]) OR "medication errors"[All Fields] OR ("medication"[All Fields] AND "error"[All Fields]) OR "medication error"[All Fields]<br><br>abuse: "abusable"[All Fields] OR "abuse's"[All Fields] OR "abused"[All Fields] OR "abuser"[All Fields] OR "abuser's"[All Fields] OR "abusers"[All Fields] OR "abuses"[All Fields] OR "abusing"[All Fields] OR "abusive"[All Fields] OR "abusively"[All Fields] OR "abusiveness"[All Fields] OR "substance-related disorders"[MeSH Terms] OR ("substance-related"[All Fields] AND "disorders"[All Fields]) OR "substance-related disorders"[All Fields] OR "abuse"[All Fields]<br><br>misuse: "misuse"[All Fields] OR "misused"[All Fields] OR "misuser"[All Fields] OR "misusers"[All Fields] OR "misuses"[All Fields] OR "misusing"[All Fields]	507,600
# 02	**Search:adverse drug reaction**<br>"drug-related side effects and adverse reactions"[MeSH Terms] OR ("drug-related"[All Fields] AND "side"[All Fields] AND "effects"[All Fields] AND "adverse"[All Fields] AND "reactions"[All Fields]) OR "drug-related side effects and adverse reactions"[All Fields] OR ("adverse"[All Fields] AND "drug"[All Fields] AND "reaction"[All Fields]) OR "adverse drug reaction"[All Fields]	175,389
# 03	**Search:opioid**<br>"analgesics, opioid"[Pharmacological Action] OR "analgesics, opioid"[MeSH Terms] OR ("analgesics"[All Fields] AND "opioid"[All Fields]) OR "opioid analgesics"[All Fields] OR "opioid"[All Fields] OR "opioids"[All Fields] OR "opioid's"[All Fields]	208,378
# 04	01 AND 02 AND 03	655

Table S3. Search strategy for Scopus database

**Search #**	**Query**	**Items found**
# 01	Search: (medication error OR abuse OR misuse)	2,916,785
# 02	Search:adverse drug reaction	271,822
# 03	Search:opioid	164,952
# 04	01 AND 02 AND 03	98

Table S4. Search strategy for EBSCO database

**Search #**	**Query**	**Items found**
# 01	Search: (medication error OR abuse OR misuse)	463.461
# 02	Search:adverse drug reaction	139,280
# 03	Search:opioid	69,455
# 04	01 AND 02 AND 03	181

Table S5. Data Extraction Template

Data Extraction Template

1.General Information

1.1 Author

1.2. Year

1.3. Country

1.4. Study design *(select from a list the appropriate type):*

- Randomised controlled trial
- Cohort study
- Cross sectional study
- Case control study
- Qualitative research
- Prevalence study
- Case series
- Case report
- Economic evaluation
- Other

2. Characteristics of Included Studies

2.1. Aim of study

2.2. Opioid substances referred in the manuscript *(select from a list the appropriate type):*

- Fentanyl
- Tramadol
- Oxycodone
- Morphine
- Buprenorphine
- Codeine
- Other

3. Participants details

3.1. Number of reports/patients (including information on percentage and total number)

3.2. Age group

4. Type of problems reported

- Misuse
- Abuse
- Medication error
- Other *(describe)*

5. Specific topics

5.1. Type of use: prescribed medicines (*Yes/No/Not stated*)

5.2. Type of use: diversion use (*Yes/No/Not stated*)

Table S6. Risk of bias evaluation of the cohort studies included in this study (n=9)

Study	Were the two groups similar and recruited from the same population?	Were the exposures measured similarly to assign people to both exposed and unexposed groups?	Was the exposure measured in a valid and reliable way?	Were confounding factors identified?	Were strategies to deal with confounding factors stated?	Were the groups/participants free of the outcome at the start of the study (or at the moment of exposure)?	Were the outcomes measured in a valid and reliable way?	Was the follow up time reported and sufficient to be long enough for outcomes to occur?	Was follow up complete, and if not, were the reasons to loss to follow up described and explored?	Were strategies to address incomplete follow up utilized?	Was appropriate statistical analysis used?	% Yes
Harris 2023	Y	Y	Y	Y	Y	Y	Y	Y	NA	NA	Y	81.82%
Tardelli 2023	Y	Y	Y	Y	Y	Y	Y	Y	U	U	Y	81.82%
Lorenzini 2022	Y	Y	Y	Y	Y	Y	Y	Y	Y	Y	Y	100%
Schutijser 2020	NA	NA	Y	NA	NA	U	Y	NA	NA	NA	Y	28%
Eluri 2018	N	NA	N	N	N	N	N	N	N	NA	Y	9.09%
Heneka 2018	NA	NA	U	N	N	NA	U	N	NA	NA	Y	9.09%
Culleré 2009	Y	Y	Y	N	N	Y	Y	NA	NA	NA	Y	55%
Boockvar 2009	NA	NA	Y	N	N	Y	Y	Y	U	N	Y	46%
Whipple 1992	NA	NA	Y	N	N	Y	Y	NA	NA	NA	NA	28%

Y=Yes N=No U=Unclear NA= Not Applicable

Table S7. Risk of bias evaluation of the case studies included in this study (n=25)

Study	1. Were there clear criteria for inclusion in the case series?	2. Was the condition measured in a standard, reliable way for all participants included in the case series?	3. Were valid methods used for identification of the condition for all participants included in the case series?	4. Did the case series have consecutive inclusion of participants?	5. Did the case series have complete inclusion of participants?	6. Was there clear reporting of the demographics of the participants in the study?	7. Was there clear reporting of clinical information of the participants?	8. Were the outcomes or follow up results of cases clearly reported?	9. Was there clear reporting of the presenting site(s)/clinic(s) demographic information?	10. Was statistical analysis appropriate?	% yes
Jobski 2023	Y	Y	Y	N	N	U	U	N	N	U	30%
Chatterton 2023	Y	Y	Y	U	U	N	N	Y	N	Y	50%
France 2023	Y	Y	Y	U	U	Y	Y	Y	U	Y	70%
Stephenson 2023	Y	Y	Y	U	U	Y	Y	Y	N	Y	70%
Gustafsson 2023	Y	Y	Y	NA	NA	U	U	Y	N	Y	50%
Ni 2023	Y	U	U	N	U	U	U	U	N	Y	20%
Chiappini 2022	Y	Y	Y	NA	NA	Y	U	U	NA	Y	50%
Andreaggi 2020	Y	N	Y	Y	Y	Y	N	U	Y	Y	70%
Schifano 2019	Y	Y	Y	N	U	Y	Y	Y	U	Y	70%
Mullan 2019	Y	Y	Y	U	U	Y	Y	Y	N	Y	70%
Gariel 2018	Y	Y	Y	Y	N	Y	Y	Y	N	Y	80%
Heneka 2018	Y	N	Y	NA	N	Y	Y	Y	N	Y	60%
Moulis 2018	Y	Y	Y	Y	Y	U	N	U	Y	NA	60%
Min 2018	Y	Y	Y	Y	Y	Y	N	U	Y	Y	80%
Seth 2018	Y	Y	Y	NA	NA	Y	NA	Y	N	Y	50%
Day 2016	Y	Y	Y	Y	Y	Y	N	U	Y	Y	80%
Beaudoin 2015	Y	Y	Y	U	U	Y	Y	Y	U	Y	70%
Lovegrove 2015	U	Y	Y	Y	Y	Y	N	U	Y	Y	70%
Brophy 2014	Y	Y	Y	Y	Y	Y	U	U	Y	Y	80%
Lövborg 2014	Y	Y	Y	Y	Y	N	N	U	Y	NA	60%
Cassidy 2011	Y	Y	Y	Y	Y	Y	N	N	y	NA	70%
Mc Donnell 2011	Y	N	Y	U	U	Y	N	N	Y	Y	50%
Bailey 2009	Y	Y	Y	Y	Y	Y	N	Y	U	N	70%
Cobaugh 2006	N	Y	Y	Y	Y	N	N	Y	Y	Y	70%
Hicks 2006	Y	Y	Y	Y	Y	N	N	U	Y	NA	60%

Y=Yes N=No U=Unclear NA= Not Applicable
